# MiR-29b suppresses the proliferation and migration of osteosarcoma cells by targeting CDK6

**DOI:** 10.1007/s13238-016-0277-2

**Published:** 2016-05-26

**Authors:** Kegan Zhu, Lei Liu, Junliang Zhang, Yanbo Wang, Hongwei Liang, Gentao Fan, Zhenhuan Jiang, Chen-Yu Zhang, Xi Chen, Guangxin Zhou

**Affiliations:** Department of Orthopedics, School of Medicine, Jinling Hospital, Nanjing University, Nanjing, 210002 China; State Key Laboratory of Pharmaceutical Biotechnology, Collaborative Innovation Center of Chemistry for Life Sciences, NJU Advanced Institute for Life Sciences (NAILS), Jiangsu Engineering Research Center for MicroRNA Biology and Biotechnology, School of Life Sciences, Nanjing University, Nanjing, 210046 China; Department of Orthopedics, The Affiliated Yixing Hospital of Jiangsu University, Yixing, 214200 China

**Keywords:** miR-29b, osteosarcoma, proliferation, migration, tumorigenesis

## Abstract

Osteosarcoma is the most common primary sarcoma of bone, and it is a leading cause of cancer death among adolescents and young adults. However, the molecular mechanism underlying osteosarcoma carcinogenesis remains poorly understood. Recently, cyclin-dependent kinase 6 (CDK6) was identified as an important oncogene. We found that CDK6 protein level, rather than CDK6 mRNA level, is much higher in osteosarcoma tissues than in normal adjacent tissues, which indicates a post-transcriptional mechanism involved in CDK6 regulation in osteosarcoma. MiRNAs are small non-coding RNAs that repress gene expression at the post-transcriptional level and have widely been shown to play important roles in many human cancers. In this study, we investigated the role of miR-29b as a novel regulator of CDK6 using bioinformatics methods. We demonstrated that CDK6 can be downregulated by miR-29b via binding to the 3′-UTR region in osteosarcoma cells. Furthermore, we identified an inverse correlation between miR-29b and CDK6 protein levels in osteosarcoma tissues. Finally, we examined the function of miR-29b-driven repression of CDK6 expression in osteosarcoma cells. The results revealed that miR-29b acts as a tumor suppressor of osteosarcoma by targeting CDK6 in the proliferation and migration processes. Taken together, our results highlight an important role for miR-29b in the regulation of CDK6 in osteosarcoma and may open new avenues for future osteosarcoma therapies.

**Electronic supplementary material:**

The online version of this article (doi:10.1007/s13238-016-0277-2) contains supplementary material, which is available to authorized users.

## Introduction

Osteosarcoma is the most common primary bone malignancy, mainly occurring in children and adolescents (Marina et al., 2004; Longhi et al., 2006). It is usually found at the end of long bones, mainly in the knee (Admassi, 2009). Osteosarcoma is the eighth leading cancer with an incidence of 4.4 per million people. Of all children diagnosed with osteosarcoma, the 5-year survival rate is less than 30%, and the 10-year survival rate is less than 50% (Marina et al., 2004; Longhi et al., 2006). Despite advances in therapeutic strategies, there is still no effective treatment for osteosarcoma due to the poor understanding of its etiology. Studies have demonstrated diverse genetic alterations in osteosarcoma cells including structural abnormalities, gain and/or loss of chromosomes, mutations in tumor suppressor genes and epigenetic modifications (Kansara and Thomas, 2007; Broadhead et al., 2011). However, the molecular mechanisms underlying the initiation, development and metastasis of this disease remain unclear. Therefore, more research should be performed to determine the molecular mechanisms underlying osteosarcoma carcinogenesis, which might provide novel strategies to improve the survival and quality of life of osteosarcoma patients.

Oncoproteins are attractive therapeutic targets as they are causally related to cancer development, and cancer cells often become dependent on them for continued proliferation and survival (Weinstein and Joe, 2006). One such oncoprotein is CDK6 (cyclin-dependent kinase 6). CDK6 is a member of the CDK family. CDKs play important roles in the major cell-cycle transitions and phases of all eukaryotic organisms either directly or indirectly. Furthermore, proliferation of mammalian cells is primarily governed by CDKs (Ekholm and Reed, 2000; Matushansky et al., 2003). CDK family members have attracted widespread attention as potential oncogenes and are widely reported to be deregulated in many cancers. Moreover, the oncogenic capacity of CDK6 has been shown in experimental models by several groups (Malumbres and Barbacid, 2009). CDK6 activation can directly lead to some of the hallmarks of cancer by causing proliferation that is independent of normal extracellular cues or by over-riding checkpoints that ensure genomic integrity and stability (Musgrove et al., 2011). Furthermore, many studies show that specific inhibitors of CDK6 have anti-tumor effects in various malignancies (Fry et al., 2001; Fry et al., 2004; Finn et al., 2009). However, little is known about the expression and function of CDK6 in osteosarcoma.

MicroRNAs (miRNAs) are a class of small, non-coding, single-stranded RNAs that bind target mRNAs at complementary sites in their 3′-untranslated regions (3′-UTRs), thereby suppressing the expression of the target gene at the post-transcriptional level in two manners: by inhibiting mRNA translation or promoting mRNA degradation (Lee et al., 2004). In animals, most miRNAs are imprecisely complementary to their mRNA targets and they inhibit protein synthesis without destroying the stability of the mRNA target (Carrington and Ambros, 2003; Ambros, 2004). Through this mechanism, miRNAs regulate a wide range of biological processes, including cell proliferation, differentiation, migration, apoptosis, development and metabolism (Calin and Croce, 2006; Ma and Weinberg, 2008; Nicoloso et al., 2009). Importantly, dysregulated and dysfunctional miRNAs play important roles in different human diseases, including most cancers, due to the ability of miRNAs to affect the translation and stability of targeted oncogenes and tumor suppressors, which eventually influences cellular physiology (Calin and Croce, 2006; Ma and Weinberg, 2008; Nicoloso et al., 2009). One such miRNA is miR-29b, the expression of which is frequently downregulated in human cancers (Mott et al., 2007; Garzon et al., 2009; Mott et al., 2010) and in osteosarcoma (Jones et al., 2012; Dai et al., 2013). In particular, it has been shown that miR-29b can function as a tumor suppressor to inhibit cancer cell proliferation (Zhang et al., 2014). However, the underlying molecular mechanisms through which downregulation of miR-29b contributes to the development and progression of osteosarcoma remain to be fully elucidated.

In this study, we found that CDK6 protein levels, but not mRNA levels, were upregulated in osteosarcoma tissues. Then, we identified CDK6 as a direct target gene of miR-29b. The inverse correlation between miR-29b and CDK6 expression level in osteosarcoma tissues and normal adjacent tissues was further analyzed. Moreover, the potential role of miR-29b as a tumor suppressor of osteosarcoma through targeting CDK6 in the processes of proliferation and migration has been experimentally validated.

## Results

### Upregulation of CDK6 protein but not mRNA in osteosarcoma tissues

**Figure 1:**
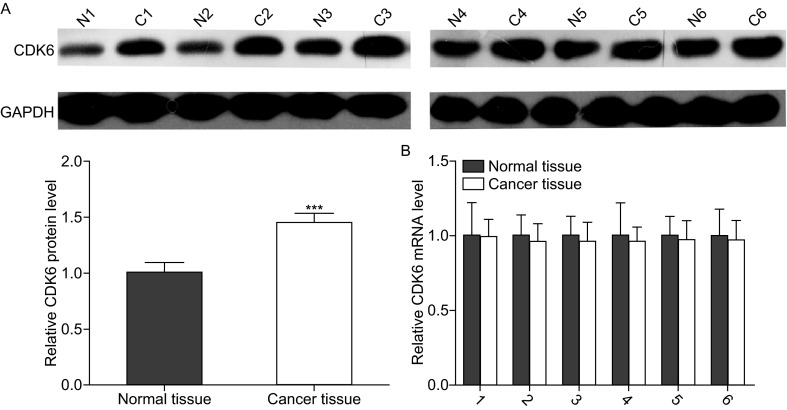
**Downregulation of CDK6 in osteosarcoma tissues**. (A) Western blotting analyses of the expression levels of the CDK6 protein in 6 pairs of osteosarcoma tissues (denoted as “C”) and corresponding noncancerous tissues (denoted as “N”). Upper panel: representative image; lower panel: quantitative analysis (***P < 0.001). (B) Quantitative RT-PCR analyses of the expression levels of CDK6 mRNA in the same 6 pairs of osteosarcoma tissues and corresponding noncancerous tissues. The results were normalized to GAPDH (***P <0.001)

We first determined the expression patterns of CDK6 in osteosarcoma tissues. After measuring the levels of CDK6 protein in 6 pairs of osteosarcoma tissues and adjacent noncancerous tissues (the clinical features of these tissue samples are listed in Table S1) via Western blotting, we found that CDK6 protein levels were significantly higher in the cancer tissues (Fig. 1A). Subsequently, we performed quantitative RT-PCR to measure the levels of CDK6 mRNA in the same 6 pairs of cancerous and noncancerous tissues. We found that CDK6 mRNA levels did not differ significantly between the cancerous and noncancerous tissues (Fig. 1B). This disparity between CDK6 protein and mRNA levels in osteosarcoma tissues strongly suggest that a post-transcriptional mechanism is involved in the regulation of CDK6.

### Identification of conserved miR-29b target sites in the 3′-UTR of CDK6

**Figure 2:**
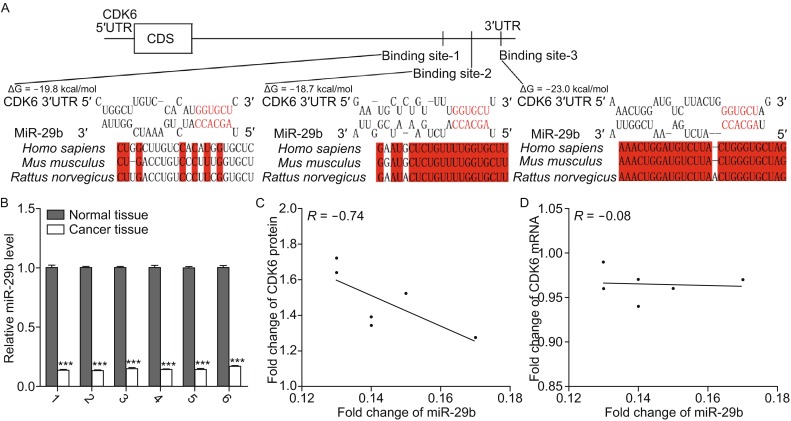
**Inverse correlation between the miR-29b and CDK6 protein expression levels in osteosarcoma tissues**. (A) Schematic description of the hypothetical duplexes formed by the interactions between the binding sites in the CDK6 3′-UTR (top) and miR-29b (bottom). The predicted free energy value of each hybrid is indicated. The seed recognition sites are denoted, and the conservation of the nucleotides in these regions across species, including human, mouse and rat, are displayed. (B) Quantitative RT-PCR analyses of the expression levels of miR-29b in the same 6 pairs of osteosarcoma tissues and corresponding noncancerous tissues. The results were normalized to U6 (***P < 0.001). (C) Pearson’s correlation scatter plot analysis of the expression levels between miR-29b and CDK6 protein in osteosarcoma tissues. (D) Pearson’s correlation scatter plot analysis of the expression levels between miR-29b and CDK6 mRNA in osteosarcoma tissues

One important mode of post-transcriptional regulation is the repression of mRNA translation by miRNAs. Therefore, miRNAs are likely to play a biologically relevant role in regulating CDK6 expression in osteosarcoma. Three computational algorithms, including TargetScan (Lewis et al., 2003), miRanda (John et al., 2004) and PicTar (Krek et al., 2005), were used in combination to identify potential miRNAs that can target CDK6. Among the numerous candidate regulatory miRNA of CDK6, we selected miR-29b for further investigation because we only focused on miRNAs that had multiple target sites within the 3′-UTR of CDK6. There were three predicted hybridizations between miR-29b and the 3′-UTR of CDK6, and the minimum free energy values of these hybridizations are −19.8, −18.7 and −23.0 kcal/mol, respectively, which are well within the range of genuine miRNA-target pairs (Fig. 2A). Moreover, there is perfect base-pairing between the seed regions (the core sequence that encompasses the first 2–7 bases of the mature miRNA) and the cognate targets. Furthermore, two of the three miR-29b binding sequences in the CDK6 3′-UTR are highly conserved across species.

### Detection of an inverse correlation between miR-29b and the CDK6 protein in osteosarcoma tissues

We next investigated whether miR-29b was inversely correlated with CDK6 in osteosarcoma. After determining the levels of miR-29b in the same 6 pairs of osteosarcoma tissues and adjacent noncancerous tissues, we found that miR-29b levels were significantly downregulated in osteosarcoma tissues (Fig. 2B). The correlation between miR-29b and CDK6 protein or mRNA levels were further illustrated using Pearson’s correlation scatter plots. The results revealed that the inverse correlation of miR-29b with the CDK6 protein (Fig. 2C) was stronger than that with the CDK6 mRNA (Fig. 2D) in the osteosarcoma tissues. Because animal miRNAs are generally believed to block translational processes without affecting transcript levels, the results strongly indicated the involvement of a miRNA-mediated post-transcriptional regulatory mechanism in CDK6 repression. In conclusion, the results of bioinformatics prediction taken together with the inverse correlation between miR-29b and CDK6 protein levels, but not mRNA levels, indicated that CDK6 is a target of miR-29b in human osteosarcoma tissues.

### Validation of CDK6 as a direct target of miR-29b

**Figure 3:**
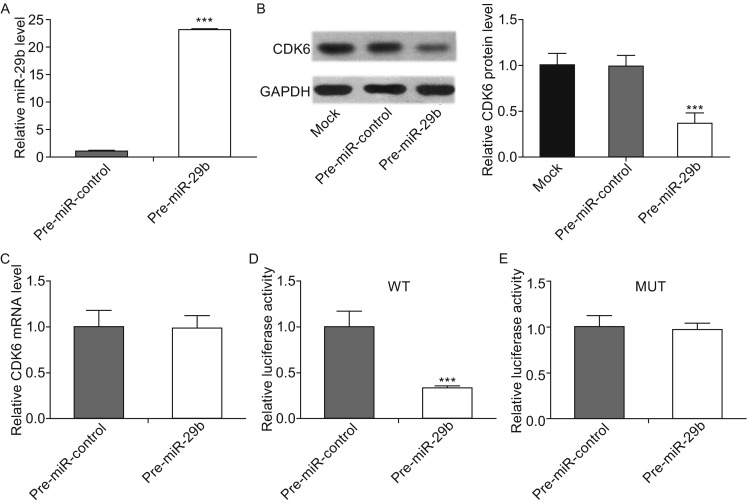
**CDK6 protein expression is inhibited by miR-29b via binding to the CDK6 3′-UTR in osteosarcoma cells**. (A) Quantitative RT-PCR analyses of the expression levels of miR-29b in osteosarcoma cells after transfection with pre-miR-29b or pre-miR-control. The results were normalized to U6 (***P < 0.001). (B) Western blotting analyses of the expression levels of CDK6 protein in osteosarcoma cells after transfection with pre-miR-29b, pre-miR-control or nothing (mock). Left panel: representative images; right panel: quantitative analysis (***P < 0.001). (C) Quantitative RT-PCR analyses of the expression levels of CDK6 mRNA in the osteosarcoma cells after transfection. The results were normalized to GAPDH. (D and E) Direct recognition and combination of the CDK6 3′-UTR by miR-29b. Firefly luciferase reporters containing either wild-type (WT) or mutant (MUT) miR-29b binding sites in the CDK6 3′-UTR were co-transfected into osteosarcoma cells with either the pre-miR-control or pre-miR-29b. Twenty-four hours post-transfection, the cells were assayed using a luciferase assay kit, and the luciferase activities were normalized to the β-galactosidase levels of the control (***P < 0.001)

The correlation between miR-29b and CDK6 was further examined by evaluating CDK6 expression in the human osteosarcoma cell line MG-63 after overexpression of miR-29b. Here, we overexpressed miR-29b by transfecting cells with pre-miR-29b, which is a synthetic RNA oligonucleotide that mimics the miR-29b precursor. The efficient overexpression of miR-29b in MG-63 cells is shown in Fig. 3A. Cellular miR-29b levels were increased approximately 25-fold when MG-63 cells were transfected with pre-miR-29b. As anticipated, overexpression of miR-29b significantly suppressed the CDK6 protein levels in MG-63 cells (Fig. 3B). Furthermore, we determined CDK6 mRNA expression levels by qRT-PCR after transfecting the cells with pre-miR-29b. As shown in Figure 3C, overexpression of miR-29b did not affect CDK6 mRNA levels in MG-63 cells. Taken together, these results demonstrated that miR-29b specifically regulates CDK6 expression at the post-transcriptional level, which is the most common mechanism for animal miRNAs.

To determine whether the negative regulatory effects of miR-29b on CDK6 expression were mediated through the binding of miR-29b to the presumed sites in the 3′-UTR of the CDK6 mRNA, a 1500 bp fragment of CDK6 3′-UTR containing the three presumed miR-29b binding sites was placed downstream of the firefly luciferase gene in a reporter plasmid. The recombinant plasmid was transfected into MG-63 cells along with pre-miR-29b or pre-miR-control. As expected, luciferase activity was markedly reduced in the cells transfected with pre-miR-29b (Fig. 3D). Furthermore, we introduced point mutations into the corresponding complementary sites in the 3′-UTR of CDK6 to eliminate the predicted miR-29b binding sites (All three binding positions were mutated). This mutated luciferase reporter was unaffected by overexpression of miR-29b (Fig. 3E). These findings suggested that the binding sites strongly contribute to the interaction between miR-29b and CDK6 mRNA. In conclusion, our results demonstrated that miR-29b directly recognizes and binds to the 3′-UTR of the CDK6 mRNA transcript and inhibits CDK6 translation in osteosarcoma cells.

### MiR-29b suppresses the proliferation of osteosarcoma cells via targeting CDK6

**Figure 4:**
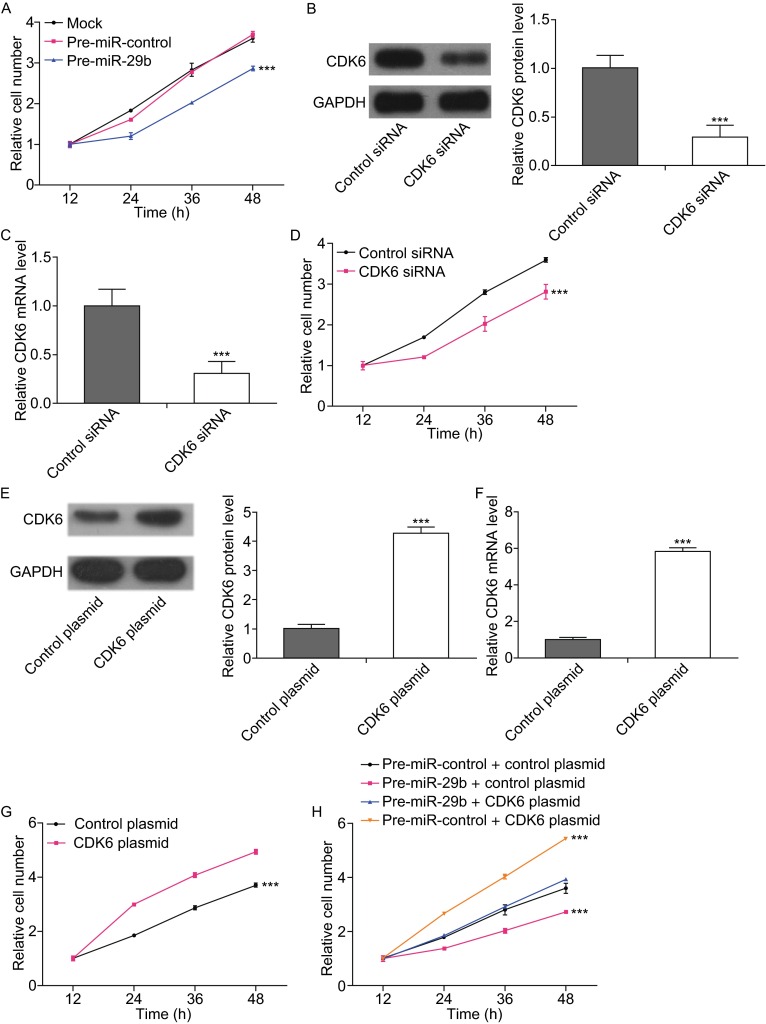
**MiR-29b represses cell proliferation via targeting CDK6 in osteosarcoma cells**. (A) The CCK8 assays were performed 12, 24, 36 and 48 h after the transfection of osteosarcoma cells with pre-miR-control, pre-miR-29b or nothing (mock). ***P < 0.001. (B) Western blotting analyses of the expression levels of the CDK6 protein in osteosarcoma cells after transfection with control siRNA or CDK6 siRNA. Left panel: representative images; right panel: quantitative analysis (***P < 0.001). (C) Quantitative RT-PCR analyses of the expression levels of CDK6 mRNA in osteosarcoma cells after transfection with control siRNA or CDK6 siRNA (***P < 0.001). (D) The CCK8 assays were performed 12, 24, 36 and 48 h after the transfection of osteosarcoma cells with control siRNA or CDK6 siRNA (***P < 0.001). (E) Western blotting analyses of the expression levels of CDK6 protein in osteosarcoma cells after transfection with control plasmid or CDK6 plasmid. Left panel: representative images; right panel: quantitative analysis (***P < 0.001). (F) Quantitative RT-PCR analyses of the expression levels of CDK6 mRNA in osteosarcoma cells after transfection with control plasmid or CDK6 plasmid (***P < 0.001). (G) The CCK8 assays were performed 12, 24, 36 and 48 h after the transfection of osteosarcoma cells with control plasmid or CDK6 plasmid (***P < 0.001). (H) The CCK8 assays were performed 12, 24, 36 and 48 h after the transfection of osteosarcoma cells with pre-miR-control plus control plasmid, pre-miR-29b plus control plasmid, pre-miR-29b plus CDK6 plasmid or pre-miR-control plus CDK6 plasmid (***P < 0.001)

We next analyzed the biological consequences of the miR-29b-driven repression of CDK6 expression in osteosarcoma cells. Because CDK6 is essential for the regulation of cell proliferation, we evaluated whether miR-29b would modulate cell proliferation via binding to CDK6 in osteosarcoma cells. First, we evaluated the effects of miR-29b on the proliferation of MG-63 cells using CCK8 assays. As expected, MG-63 cells transfected with pre-miR-29b showed decreased proliferation (Fig. 4A). Subsequently, we investigated the role of CDK6 on cell proliferation by overexpression or knockdown of CDK6 to provide a better understanding of the CDK6-involved pathway in osteosarcoma. To knock down CDK6, a siRNA targeting CDK6 was designed and then transfected into MG-63 cells. Both CDK6 protein (Fig. 4B) and mRNA (Fig. 4C) levels were significantly reduced by CDK6 siRNA. Then, we performed CCK8 assays to determine cell proliferation ability after transfection with control siRNA or CDK6 siRNA. Similar to miR-29b overexpression, transfection of CDK6 siRNA markedly reduced the cell proliferation ability of MG-63 cells (Fig. 4D). To overexpress CDK6, an expression plasmid designed to specifically express the full-length open reading frame (ORF) of CDK6 without the miR-29b-responsive 3′-UTR was constructed and transfected into MG-63 cells. Both CDK6 protein (Fig. 4E) and mRNA (Fig. 4F) levels were significantly increased by the CDK6 plasmid. Then, we performed CCK8 assays to determine cell proliferation ability after transfection with the control plasmid or CDK6 plasmid. Consistent with previous studies showing that CDK6 functions as a proliferation promoter, transfection of the CDK6 plasmid markedly increased the cell proliferation ability of MG-63 cells (Fig. 4G). Moreover, compared with cells transfected with pre-miR-29b alone, those transfected with both pre-miR-29b and the CDK6-ORF-overexpression plasmid exhibited significantly higher proliferation rates (Fig. 4H), suggesting that miR-29b-resistant CDK6 is sufficient to rescue the suppression of CDK6 by miR-29b and to attenuate the anti-proliferation effect of miR-29b on osteosarcoma cells.

### MiR-29b suppresses the migration ability of osteosarcoma cells via targeting CDK6

**Figure 5:**
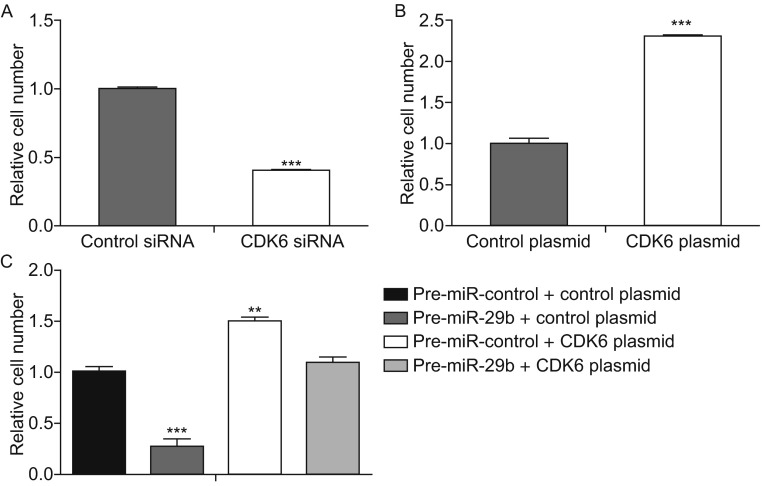
**MiR-29b represses cell migration via targeting CDK6 in osteosarcoma cells**. (A) Transwell analyses of the migrated osteosarcoma cells after transfection with the control siRNA or CDK6 siRNA (***P < 0.001). (B) Transwell analyses of the migrated osteosarcoma cells after transfection with the control plasmid or CDK6 plasmid (***P < 0.001). (C) Transwell analyses of the migrated osteosarcoma cells after transfection with pre-miR-control plus control plasmid, pre-miR-29b plus control plasmid, pre-miR-29b plus CDK6 plasmid or pre-miR-control plus CDK6 plasmid (**P < 0.01; ***P < 0.001)

To further test the biological effect of CDK6-targeted miR-29b on osteosarcoma cells, a series of Transwell assays were performed to determine the cell migration ability. As expected, MG-63 cells transfected with CDK6 siRNA showed inhibited cell migration (Fig. 5A). In contrast, transfection with the CDK6-overexpression plasmid had the opposite effect on cell migration (Fig. 5B). Furthermore, cells transfected with pre-miR-29b alone displayed repressed migration ability (Fig. 5C). When MG-63 cells were simultaneously transfected with pre-miR-29b and the CDK6 overexpression plasmid, CDK6 dramatically attenuated the migration suppression by miR-29b (Fig. 5C). Taken together, these results demonstrate that miR-29b inhibits cell migration by silencing CDK6.

## Discussion

Osteosarcoma is the most common primary sarcoma of bone, and it is a leading cause of cancer death among adolescents and young adults (Ottaviani and Jaffe, 2009). The mechanisms that initiate and propagate osteosarcoma genesis remain poorly understood (Gorlick, 2009). CDK6 is a protein that initiates the release of the RB-dependent cell cycle-inhibitory ‘brake’ that governs cell cycle transitions during quiescence, senescence and differentiation (Musgrove et al., 2011). Furthermore, as an oncogene, CDK6 can promote tumor cell proliferation and is widely deregulated in different cancers, such as breast cancer, glioma, blastoma, lymphoma and melanoma (Malumbres and Barbacid, 2001; Malumbres and Barbacid, 2009). Some inhibitors of the cyclin D-associated kinases CDK4 and CDK6 may be used as potential cancer therapeutics. However, the function and regulation of CDK6 in osteosarcoma is still largely unknown. In this study, we first showed that CDK6 protein levels were significantly higher in 6 pairs of osteosarcoma tissues than in corresponding noncancerous tissues. Furthermore, we found that silencing CDK6 expression using siRNA could inhibit proliferation and migration of osteosarcoma cells, whereas overexpressing CDK6 induced opposing effects, validating its role as an essential oncogene during osteosarcoma tumorigenesis. Interestingly, CDK6 mRNA levels in human osteosarcoma tissues were not significantly different from CDK6 mRNA levels in corresponding noncancerous tissues. These results suggest a post-transcriptional regulation mechanism involved in CDK6 repression. One centrally important mode of post-transcriptional regulation is the repression of mRNA transcripts by miRNAs. Therefore, we searched for miRNAs that can target CDK6 and identified miR-29b as a novel candidate. In addition, by overexpressing miR-29b in osteosarcoma cells, we experimentally validated the direct inhibition of CDK6 translation by miR-29b. Finally, we showed that miR-29b inhibited CDK6 expression and consequently inhibited proliferation and migration in cultured osteosarcoma cells. Our studies reveal the importance of miR-29b targeting CDK6 as a novel regulatory pathway in osteosarcoma progression.

MiRNAs are aberrantly expressed in cancers and can function as oncogenes or tumor suppressor genes (Calin and Croce, 2006; Esquela-Kerscher and Slack, 2006). In this study, we found that the levels of miR-29b were lower in osteosarcoma tissues than in noncancerous tissues. These results suggest that miR-29b may be involved in the pathogenesis of osteosarcoma as a tumor suppressor. Indeed, miR-29b has been reported to be downregulated in several types of human cancer, including hepatocellular carcinoma (Fang et al., 2011), myeloid leukemia (Mott et al., 2010) and chronic lymphocytic leukemia (Sampath et al., 2012). Furthermore, miR-29b plays a tumor suppressive role in cancer by influencing cell survival, tumor growth, apoptosis, cell cycle distribution, migration and angiogenesis (Cortez et al., 2010; Fang et al., 2011; Kole et al., 2011; Wang et al., 2011; Rossi et al., 2013). In this study, we found that overexpressing miR-29b can inhibit proliferation and migration of osteosarcoma cells and that CDK6 reduction can mimic the effect of miR-29b induction. Interestingly, we observed that the restoration of CDK6 expression can successfully attenuate the anti-proliferation and anti-migration effects of miR-29b on osteosarcoma cells, although miR-29b has many other targets. These results suggest that the targeting of CDK6 is a major mechanism by which miR-29b exerts its tumor-suppressive function.

Taken as a whole, this study delineates a novel regulatory network employing miR-29b and CDK6 to regulate proliferation and migration in osteosarcoma cells. Considering that re-expression of miRNAs that are lost in cancers, through either transfection or viral delivery method, has been demonstrated to be potential therapeutic method against human cancers (Kumar et al., 2008; Iorio and Croce, 2012), our study may open new avenues for future osteosarcoma therapies.

## Materials and methods

### Cells and human tissues

The human osteosarcoma cell line MG-63 was purchased from the Shanghai Institute of Cell Biology, Chinese Academy of Sciences (Shanghai, China). MG-63 cells were cultured in DMEM supplemented with 10% fetal bovine serum (GIBCO, CA, USA) and incubated in 5% CO_2_ at 37°C in a water-saturated atmosphere. The osteosarcoma and paired normal adjacent tissues were derived from patients undergoing a surgical procedure at the Jinling Hospital of Nanjing University (Nanjing, China). All protocols concerning the use of patient samples in this study were approved by the Medical Ethics Committee of the Jinling Hospital of Nanjing University (Nanjing, China). A signed consent form was obtained from each donor. The tissue fragments were immediately frozen in liquid nitrogen at the time of surgery and stored at −80°C. The clinical features of the patients are listed in Table S1. The study protocol was approved by the Medical Ethics Committee of the Jinling Hospital of Nanjing University (Nanjing, China), and all experiments were performed in accordance with approved guidelines of the Jinling Hospital of Nanjing University (Nanjing, China).

### RNA isolation and quantitative RT-PCR

Total RNA was extracted from the cultured cells and human tissues using TRIzol Reagent (Invitrogen, Carlsbad, CA) according to the manufacturer’s instructions. Assays to quantify miRNAs were performed using Taqman miRNA probes (Applied Biosystems, Foster City, CA) according to the manufacturer’s instructions. Briefly, 1 µg of total RNA was reverse-transcribed to cDNA using AMV reverse transcriptase (TaKaRa, Dalian, China) and a stem-loop RT primer (Applied Biosystems). The reaction conditions were as follows: 16°C for 30 min, 42°C for 30 min, and 85°C for 5 min. Real-time PCR was performed using a TaqMan PCR kit on an Applied Biosystems 7300 Sequence Detection System (Applied Biosystems). The reactions were incubated in a 96-well optical plate at 95°C for 5 min, followed by 40 cycles of 95°C for 15 s and 60°C for 1 min. All of the reactions were run in triplicate. After the reaction, the cycle threshold (C_T_) data were determined using fixed threshold settings, and the mean C_T_ of the triplicate PCRs was determined. A comparative C_T_ method was used to compare each condition to the controls. The relative levels of the miRNAs in cells and tissues were normalized to U6. The amount of miRNA relative to the internal control U6 was calculated using the 2^−∆∆CT^ equation, in which ∆∆C_T_ = (C_T miRNA_ − C_T U6_) _test condition_ − (C_T miRNA_ − C_T U6_) _control condition_.

To quantify CDK6 mRNA, 1 µg of total RNA was reverse-transcribed to cDNA using oligo dT and AMV reverse transcriptase (TaKaRa) in the reaction, which was performed with the following conditions: 42°C for 60 min and 70°C for 10 min. Next, real-time PCR was performed using the RT product, SYBER Green Dye (Invitrogen), and specific primers for CDK6 and GAPDH. The sequences of the primers were as follows: CDK6 (sense): 5′-TGCACAGTGTCACGAACAGA-3′; CDK6 (antisense): 5′-ACCTCGGAGAAGCTGAAACA-3′; GAPDH (sense): 5′-GATATTGTTGCCATCAATGAC-3′; and GAPDH (antisense): 5′-TTGATTTTGGAGGGATCTCG-3′. The reactions were incubated at 95°C for 5 min, followed by 40 cycles of 95°C for 30 s, 60°C for 30 s, and 72°C for 30 s. After the reactions were complete, the C_T_ values were determined by setting a fixed threshold. The relative amount of CDK6 mRNA was normalized to GAPDH.

### Cell transfection

Synthetic pre-miR-29b and scrambled negative control RNAs (pre-miR-control) were purchased from Ambion (Austin, TX, USA). Cells were seeded in 6-well plates and were transfected using Lipofectamine 2000 (Invitrogen) the following day when the cells were approximately 70% confluent. In each well, equal amounts of pre-miR-29b or scrambled negative control RNA were used. The cells were harvested 48 h after transfection for quantitative RT-PCR and Western blotting.

### Luciferase reporter assay

To test the direct binding of miR-29b to the target gene CDK6, a luciferase reporter assay was performed as previously described (Chen et al., 2009). Briefly, a 1500 bp fragment of human CDK6 3′-UTR containing the three presumed miR-29b binding sites was directly synthesized by Realgene (Nanjing, China). The synthetic product was inserted into the p-MIR-reporter plasmid (Ambion), and the insertion was confirmed by sequencing. To test the binding specificity, a 1500 bp fragment of mutant CDK6 3′-UTR containing three mutant miR-29b binding sites was synthesized and inserted into an equivalent luciferase reporter. For luciferase reporter assays, MG-63 cells were cultured in 12-well plates, and each well was transfected with 0.8 µg of firefly luciferase reporter plasmid, 0.8 µg of a β-galactosidase (β-gal) expression plasmid (Ambion), and equal amounts (40 pmol) of pre-miR-29b or the scrambled negative control RNA using Lipofectamine 2000 (Invitrogen). The β-gal plasmid was used as a transfection control. Twenty-four hours after transfection, the cells were assayed using a luciferase assay kit (Promega, Madison, WI, USA).

### Plasmid construction and siRNA interference assay

The siRNA sequence targeting the human CDK6 cDNA was designed and synthesized by GenePharma (Shanghai, China). The siRNA sequence was 5′-TACTTCTGAAGTGTTTGACATTT-3′. A scrambled siRNA was included as a negative control. A mammalian expression plasmid encoding the human CDK6 open reading frame (pReceiver-M02-CDK6) was purchased from GeneCopoeia (Germantown, MD, USA). An empty plasmid served as a negative control. The CDK6 expression plasmid and CDK6 siRNA were transfected into MG-63 cells using Lipofectamine 2000 (Invitrogen) according to the manufacturer’s instructions. Total RNA and protein were isolated 48 h post-transfection. The CDK6 mRNA and protein expression levels were assessed by quantitative RT-PCR and Western blotting.

### Protein extraction and Western blotting

All cells were rinsed with PBS (pH 7.4) and lysed on ice for 30 min in RIPA lysis buffer (Beyotime, China) supplemented with a Protease and Phosphatase Inhibitor Cocktail (Thermo Scientific 78440). The tissue samples were snap frozen with liquid nitrogen, ground into powder, and lysed on ice for 30 min in RIPA lysis buffer containing the Protease and Phosphatase Inhibitor Cocktail. When necessary, sonication was used to facilitate lysis. Cell lysates or tissue homogenates were centrifuged for 10 min (at 12,000 ×g and 4°C). The supernatant was collected, and the protein concentration was calculated using the Pierce BCA protein assay kit (Thermo Scientific, Rockford, IL, USA). The protein levels were analyzed via Western blot using the corresponding antibodies. The protein levels were normalized by probing the same blots with a GAPDH antibody. The following antibodies were purchased from the corresponding sources: anti-CDK6 (Santa Cruz Biotechnology sc-130545, Santa Cruz, CA, USA) and anti-GAPDH (Santa Cruz Biotechnology sc-365062, Santa Cruz, CA, USA). Protein bands were analyzed using ImageJ software (National Institutes of Health, USA).

### Cell proliferation assay

The transfected cells were seeded in 96-well plates at a density of 5 × 10^3^ cells per well. The cell proliferation index was measured using a CCK8 assay (Dojindo, Japan) at 12, 24, 36 and 48 h after the cells were seeded. Five wells were tested for each group at each time point. A volume of 10 µL CCK8 solution from the CCK8 kit was added to each well. After incubation for 2 h, plates were read at a wavelength of 450 nm to measure the absorbance of each well. The day when seeding was practiced was set as Day 0, and the relative cell number was calculated by the ratio of absorbance of Day n to Day 0.

### Cell migration assay

The MG-63 cells were transfected as described above, and the migration ability was tested in a Transwell Boyden Chamber (6.5-mm, Costar, USA). The polycarbonate membranes (8-µm pore size) on the bottom of the upper compartment of the Transwells were coated with 1% human fibronectin (R&D systems 1918-FN, USA). The cells were harvested 12 h after transfection and suspended in FBS-free DMEM. Then, cells were added to the upper chamber (4 × 10^4^ cells/well). At the same time, 0.6 mL of DMEM containing 10% FBS was added to the lower compartment, and the Transwell-containing plates were incubated for 12 h in a 5% CO_2_ atmosphere saturated with H_2_O. After incubation, cells that had entered the lower surface of the filter membrane were fixed with 4% paraformaldehyde for 20 min at room temperature, washed 3 times with PBS, and stained with 0.1% crystal violet in 0.1 mol/L borate and 2% ethanol for 15 min at room temperature. Cells remaining on the upper surface of the filter membrane (non-migrant) were gently scraped off using a cotton swab and the cells on the lower surface were counted randomly in 3 scopes at least.

### Statistical analysis

All Western blot images are representative of at least three independent experiments. Quantitative RT-PCR, luciferase reporter assays, and cell proliferation and migration assays were performed in triplicate, and each experiment was repeated several times. The data shown are the mean ± SE of at least three independent experiments. The differences were considered statistically significant at *P* < 0.05 using Student’s *t*-test.

## Electronic supplementary material

Below is the link to the electronic supplementary material.

Supplementary material 1 (PDF 66 kb)
