# Supplementary material for: MiR-29b suppresses the proliferation and migration of osteosarcoma cells by targeting CDK6
Source: Protein Cell. 2016 May 26;7(6):434–44. doi: 10.1007/s13238-016-0277-2 (PMC4887333; doi:10.1007/s13238-016-0277-2)
Supplement: Supplementary file 1 — Supplementary material 1 (PDF 66 kb) [file 13238_2016_277_MOESM1_ESM.pdf]

Table S1. Summary of clinic characteristics of patients with osteosarcoma

	Clinic characteristics	Number of cases
**Age**	≥15	5
	<15	1
**Gender**	Male	3
	Female	3
**Location**	Femur	2
	Tibia	2
	Humeral bone	1
	other	1
